# Redetermination and invariom refinement of 1-cyclo­propyl-6-fluoro-4-oxo-7-(piperazin-4-ium-1-yl)-1,4-dihydro­quinoline-3-carboxyl­ate hexa­hydrate at 120 K

**DOI:** 10.1107/S1600536808037409

**Published:** 2008-11-13

**Authors:** Francesca P. A. Fabbiani, Birger Dittrich

**Affiliations:** a Georg-August Universität Göttingen, GZG, Abteilung Kristallographie, Goldschmidtstrasse 1, 37077 Göttingen, Germany; b Georg-August Universität Göttingen, Institut für Anorganische Chemie, Tammannstrasse 4, 37077 Göttingen, Germany

## Abstract

The structure of the title compound, C_17_H_18_FN_3_O_3_·6H_2_O, has been redetermined at 120 K. An invariom refinement, a structural refinement using aspherical scattering factors from theoretically predicted multipole population parameters, yields accurate geometry and anisotropic displacement parameters, including hydrogen-bonding parameters. All potential hydrogen-bond donors and acceptors are involved in hydrogen bonding, forming an intricate three-dimensional network of N—H⋯O and O—H⋯O bonds.

## Related literature

For related literature on the invariom refinement procedure, see: Dittrich *et al.* (2005 ▶); Hübschle *et al.* (2007 ▶); Hansen & Coppens (1978 ▶). For the original structure determination and background information on quinolone anti­bacterial agents, see: Turel *et al.* (1997 ▶); Turel (2002 ▶); Mitscher (2005 ▶).
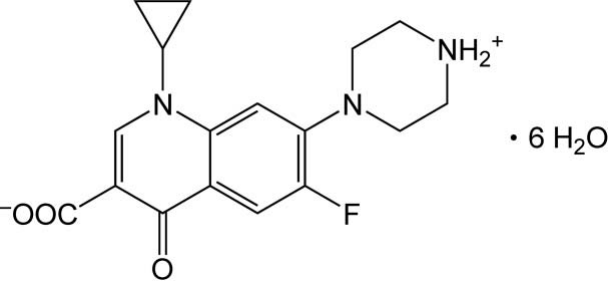


## Experimental

### 

#### Crystal data

C_17_H_18_FN_3_O_3_·6H_2_O
*M* *_r_* = 439.44
Triclinic, 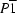

*a* = 9.5079 (3) Å
*b* = 9.9437 (3) Å
*c* = 11.0391 (3) Å
α = 94.227 (2)°
β = 100.206 (2)°
γ = 91.327 (2)°
*V* = 1023.66 (6) Å^3^
*Z* = 2.0
Mo *K*α radiation
μ = 0.12 mm^−1^
*T* = 120 K
0.30 × 0.25 × 0.03 mm

#### Data collection

Bruker APEXII diffractometer
Absorption correction: multi-scan (*SADABS*; Sheldrick, 1996 ▶) *T*_min_ = 0.918, *T*_max_ = 0.996
35927 measured reflections
7766 independent reflections
6705 reflections with *F* > 3σ(*F*)
*R*_int_ = 0.037

#### Refinement

*R*[*F* ^2^ > 2σ(*F* ^2^)] = 0.024
*wR*(*F* ^2^) = 0.032
*S* = 2.09
6705 reflections
391 parameters
All H-atom parameters refined
Δρ_max_ = 0.22 e Å^−3^
Δρ_min_ = −0.29 e Å^−3^

### 

Data collection: *APEX2* (Bruker, 2007 ▶); cell refinement: *SAINT* (Bruker, 2007 ▶); data reduction: *SAINT*; method used to solve structure: from known coordinates (Turel *et al.*, 1997 ▶); program(s) used to refine structure: *CRYSTALS* (Betteridge *et al.*, 2003 ▶) and *XD* (Koritsánszky *et al.*, 2003 ▶); molecular graphics: *ORTEPIII* (Burnett & Johnson, 1996 ▶); software used to prepare material for publication: *XDCIF* (Koritsánszky *et al.*, 2003 ▶) and *publCIF* (Westrip, 2008 ▶).

## Supplementary Material

Crystal structure: contains datablocks global, I. DOI: 10.1107/S1600536808037409/bi2314sup1.cif

Structure factors: contains datablocks I. DOI: 10.1107/S1600536808037409/bi2314Isup2.hkl

Additional supplementary materials:  crystallographic information; 3D view; checkCIF report

## Figures and Tables

**Table 1:** Hydrogen-bond geometry (Å, °)

*D*—H⋯*A*	*D*—H	H⋯*A*	*D*⋯*A*	*D*—H⋯*A*
N3—H311⋯O51^i^	1.02 (1)	2.53 (1)	3.0458 (8)	110 (1)
N3—H311⋯O41^ii^	1.02 (1)	1.98 (1)	2.8063 (7)	136 (1)
N3—H312⋯O91	1.02 (1)	1.81 (1)	2.8153 (7)	165 (1)
O41—H412⋯O1	0.96 (1)	1.84 (1)	2.8064 (6)	175 (1)
O41—H411⋯O81^iii^	0.93 (1)	1.89 (1)	2.8055 (8)	168 (1)
O51—H511⋯O81^iii^	0.96 (1)	1.87 (1)	2.8032 (8)	163 (1)
O51—H512⋯O1	0.90 (1)	1.94 (1)	2.8265 (7)	167 (1)
O61—H611⋯O71^iv^	0.95 (1)	1.87 (1)	2.8150 (8)	172 (1)
O61—H612⋯O71^v^	0.97 (1)	1.93 (1)	2.8842 (8)	168 (1)
O71—H711⋯O2	0.94 (1)	2.04 (1)	2.8392 (7)	141 (1)
O71—H711⋯O3	0.94 (1)	2.30 (1)	3.0702 (7)	138 (1)
O71—H712⋯O51^vi^	0.92 (1)	1.92 (1)	2.8272 (8)	166 (1)
O81—H811⋯O2^vii^	0.90 (1)	2.05 (1)	2.8624 (7)	149 (1)
O81—H811⋯O3^vii^	0.90 (1)	2.50 (1)	3.1874 (7)	133 (1)
O81—H812⋯O3	0.95 (1)	1.76 (1)	2.7104 (7)	172 (1)
O91—H911⋯O61	0.92 (1)	1.91 (1)	2.8085 (7)	168 (1)
O91—H912⋯O2^v^	0.94 (1)	1.79 (1)	2.7102 (6)	164 (1)

Symmetry codes: (i) 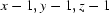; (ii) 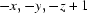; (iii) 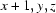; (iv) 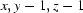; (v) 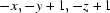; (vi) 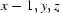; (vii) 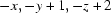.

**Table 2:** Invarioms and model compounds used for aspherical refinement of the title compound

Atom label	Invariom assigned	Model compound
F1	F1c	fluoromethane
O1, O2, O3	O1.5c[1.5o1c]−	acetic acid anion
O41–O91	O1h1h	water
N1, N2	N1c1c1c	trimethylamine
N3	N1c1c1h1h+	*N*,*N*-dimethylammonium
C1	C1n1c1c1h	2-aminopropane
C2, C3	C1c1c1h1h	propane
C4	C1.5c[1.5c1c]1.5c[1.5c1h]1n	*o*-methylaniline
C5	C1.5n[1.5c1c]1.5c[1.5c1c]1h+	*N*-meth­yl-3-methylpyridinium
C6	C1.5c[1.5n1h]1.5c[1.5c1o]1c+	3-meth­yl-4-hydroxypyridinium
C7	C1.5o1.5o1c−	acetic acid anion
C8	C2o1c1c	acetone
C9	C1.5c[1.5c1n]1.5c[1.5c1h]1c	*o*-methylaniline
C10	C1.5c[1.5c1f]1.5c[1.5c1c]1h	1-fluoro-3-methylbenzene
C11	C1.5c[1.5c1n]1.5c[1.5c1h]1f	2-fluoroaniline
C12	C1.5c[1.5c1f]1.5c[1.5c1h]1n	2-fluoroaniline
C13–C16	C1n1c1h1h	aminoethane
C17	C1.5c[1.5c1n]1.5c[1.5c1n]1h	*m*-phenylenediamine
H312, H322	H1n[1c1c1h]+	dimethylammonium
H11	H1c[1n1c1c]	2-aminopropane
H21–H32	H1c[1c1c1h]	propane
H51	H1c[1.5n1.5c]	pyridine
H101, H171	H1c[1.5*c*1.5c]	benzene
H131–H162	H1c[1n1c1h]	aminoethane
H411–H912	H1o[1h]	water

**Fig. 1.**
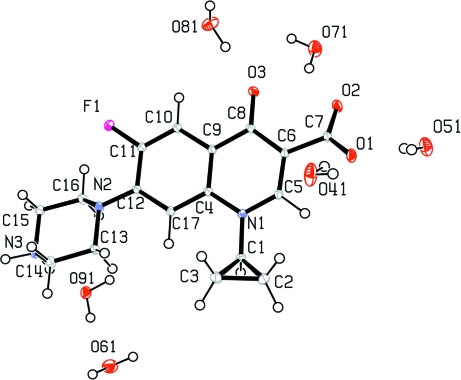
The title compound with displacement ellipsoids drawn at the 50% probability level. H atoms are shown as spheres of arbitrary radius.

## supplementary crystallographic information

### Comment

The title compound, commonly known as ciprofloxacin hexahydrate, belongs to the quinolone family of synthetic antibiotics (Turel, 2002; Mitscher, 2005). In this study, the structure of ciprofloxacin hexahydrate (Fig. 1), has been redetermined at 120 K using a 30 W microfocus Mo sealed tube. An invariom refinement (Dittrich *et al.*, 2005), a structural refinement using aspherical scattering factors from theoretically predicted multipole population parameters, yields accurate ADPs and molecular geometries, including hydrogen-bonding parameters. All primary bond lengths and angles are in good agreement with those of the previously reported room-temperature structure (Turel *et al.*,1997), but are more precise. The crystal structure exhibits an intricate 3-D hydrogen-bonding pattern. All potential hydrogen-bond donors and acceptors are involved in hydrogen bonding: water O41, O61 and O91 accept one hydrogen bond; O51, O71, O81 and carboxyl O1 accept two; both carboxyl O2 and carbonyl O3 accept three. The majority of hydrogen bonds are linear; N3—H311, O71—H711 and O8—H811 form bifurcated ones.

### Experimental

Ciprofloxacin (Sigma Aldrich) was used as received. Single crystals suitable for X-ray measurements were obtained by recrystallization from water by slow evaporation at room temperature.

### Refinement

The refinement was initiated with the original structure determined at ambient temperature by Turel *et al.* (1997) (CSD refcode COVPIN). We note that the numbering scheme in the original paper differs from that of the deposited structure. We used the same numbering scheme as in COVPIN. We also note that although the original paper reports refined H atom positions, these are not present in the deposited structure. An Independent Atom Model (IAM) refinement with *CRYSTALS* (Betteridge *et al.*, 2003) provided starting values for subsequent invariom refinement (Dittrich *et al.*, 2005), which is based on the Hansen & Coppens multipole formalism (Hansen & Coppens, 1978). This non-spherical atom refinement, which included reflections with [*F* > 3 σ(*F*)], was performed with *XDLSM* as included in the *XD* package (Koritsánszky *et al.*, 2003). *XD* input files were processed with the program *InvariomTool* (Hübschle *et al.*, 2007). For invariom refinement, non-spherical valence scattering contributions for atoms in an environment of simple bonds were obtained from theoretical calculations on model compounds that included nearest-neighbour atoms, whereas for H-atoms and atoms in a delocalized chemical environment, model compounds also included the next-nearest neighbour atoms (see table in the Supplementary Information). Full details for the general invariom modelling procedure of organic molecules can be found in Hübschle *et al.* (2007). Since in the invariom refinement the multipole parameters are fixed at theoretically predicted values, only the positional and displacement parameters were refined. Bond distances to H-atoms were freely refined due to the high quality of the data set, but can optionally be set to the values found in the model compounds, which are very close to those obtained from neutron diffraction.

### Crystal data

**Table:**

C_17_H_18_FN_3_O_3_·6H_2_O	*Z* = 2.0
*M**_r_* = 439.44	*F*_000_ = 468
Triclinic, *P*1	*D*_x_ = 1.426 Mg m^−^^3^
Hall symbol: -P 1	Mo *K*α radiation λ = 0.71073 Å
*a* = 9.5079 (3) Å	Cell parameters from 9906 reflections
*b* = 9.9437 (3) Å	θ = 2.6–35.8º
*c* = 11.0391 (3) Å	µ = 0.12 mm^−^^1^
α = 94.227 (2)º	*T* = 120 K
β = 100.206 (2)º	Plate, colourless
γ = 91.327 (2)º	0.30 × 0.25 × 0.03 mm
*V* = 1023.66 (6) Å^3^	

### Data collection

**Table:**

Bruker APEXII diffractometer	7766 independent reflections
Radiation source: Mo microsource	6705 reflections with *F* > 3σ(*F*)
Monochromator: graphite	*R*_int_ = 0.037
*T* = 120 K	θ_max_ = 33.1º
ω scans	θ_min_ = 2.1º
Absorption correction: multi-scan(SADABS; Sheldrick, 1996)	*h* = −14→14
*T*_min_ = 0.918, *T*_max_ = 0.996	*k* = −15→15
35927 measured reflections	*l* = 0→16

### Refinement

**Table:**

Refinement on *F*	Secondary atom site location: difference Fourier map
Least-squares matrix: full	Hydrogen site location: difference Fourier map
*R*[*F*^2^ > 2σ(*F*^2^)] = 0.024	All H-atom parameters refined
*wR*(*F*^2^) = 0.032	*w*1 = 1/[s^2^(*F*_o_)]
*S* = 2.09	(Δ/σ)_max_ < 0.001
6705 reflections	Δρ_max_ = 0.22 e Å^−^^3^
391 parameters	Δρ_min_ = −0.29 e Å^−^^3^
Primary atom site location: none	Extinction correction: none

### Fractional atomic coordinates and isotropic or equivalent isotropic displacement parameters (Å^2^)

**Table:**

	*x*	*y*	*z*	*U*_iso_*/*U*_eq_	
F(1)	−0.33045 (4)	0.12852 (4)	0.58299 (3)	0.017	
O(1)	0.40535 (4)	0.62476 (4)	0.78894 (4)	0.016	
O(2)	0.20874 (4)	0.70663 (4)	0.84664 (4)	0.017	
O(3)	−0.01084 (5)	0.50635 (4)	0.80861 (4)	0.018	
O(41)	0.51207 (6)	0.36492 (5)	0.80865 (6)	0.032	
O(51)	0.67335 (6)	0.70056 (6)	0.93720 (5)	0.026	
O(61)	−0.12179 (6)	0.06543 (6)	−0.12561 (5)	0.028	
O(71)	−0.05227 (6)	0.79577 (5)	0.91011 (5)	0.027	
O(81)	−0.21443 (5)	0.44232 (6)	0.94044 (5)	0.024	
O(91)	−0.35014 (5)	0.08636 (5)	0.00415 (4)	0.017	
N(1)	0.17346 (5)	0.36898 (5)	0.51490 (4)	0.011	
N(2)	−0.22506 (5)	0.04178 (5)	0.37452 (4)	0.011	
N(3)	−0.37382 (5)	−0.12031 (5)	0.16130 (5)	0.012	
C(1)	0.23877 (6)	0.33110 (6)	0.40859 (5)	0.012	
C(2)	0.39731 (6)	0.31298 (7)	0.42941 (6)	0.018	
C(3)	0.29514 (6)	0.19203 (6)	0.39590 (6)	0.016	
C(4)	0.04233 (5)	0.31170 (5)	0.52812 (5)	0.01	
C(5)	0.23769 (6)	0.46656 (5)	0.59958 (5)	0.011	
C(6)	0.18357 (6)	0.51677 (5)	0.70044 (5)	0.011	
C(7)	0.27219 (6)	0.62394 (5)	0.78547 (5)	0.012	
C(8)	0.04741 (6)	0.46515 (6)	0.72033 (5)	0.012	
C(9)	−0.02011 (5)	0.35741 (5)	0.62955 (5)	0.01	
C(10)	−0.14985 (6)	0.29436 (6)	0.64428 (5)	0.012	
C(11)	−0.21170 (6)	0.19255 (6)	0.56113 (5)	0.012	
C(12)	−0.15499 (6)	0.14755 (5)	0.45515 (5)	0.011	
C(13)	−0.14735 (6)	−0.01330 (6)	0.27971 (5)	0.012	
C(14)	−0.22196 (6)	−0.14286 (6)	0.21485 (5)	0.013	
C(15)	−0.44960 (6)	−0.06561 (6)	0.26046 (5)	0.013	
C(16)	−0.37577 (6)	0.06451 (6)	0.32136 (5)	0.012	
C(17)	−0.02664 (6)	0.20945 (5)	0.44071 (5)	0.011	
H(311)	−0.4250 (10)	−0.2100 (10)	0.1250 (10)	0.043 (3)	
H(312)	−0.3779 (9)	−0.0550 (9)	0.0933 (9)	0.027 (2)	
H(11)	0.1918 (9)	0.3733 (8)	0.3296 (8)	0.029 (2)	
H(21)	0.4516 (9)	0.3257 (8)	0.5233 (8)	0.029 (2)	
H(22)	0.4485 (9)	0.3462 (9)	0.3602 (9)	0.037 (2)	
H(31)	0.2831 (9)	0.1433 (9)	0.3068 (9)	0.033 (2)	
H(32)	0.2849 (9)	0.1304 (9)	0.4675 (8)	0.035 (2)	
H(51)	0.3399 (9)	0.5067 (9)	0.5852 (7)	0.028 (2)	
H(101)	−0.1957 (9)	0.3228 (9)	0.7198 (8)	0.031 (2)	
H(131)	−0.0424 (9)	−0.0394 (9)	0.3266 (8)	0.029 (2)	
H(132)	−0.1367 (9)	0.0571 (9)	0.2100 (8)	0.028 (2)	
H(141)	−0.1715 (9)	−0.1792 (9)	0.1422 (8)	0.030 (2)	
H(142)	−0.2228 (9)	−0.2200 (9)	0.2761 (8)	0.028 (2)	
H(151)	−0.4442 (9)	−0.1406 (9)	0.3245 (8)	0.034 (2)	
H(152)	−0.5590 (10)	−0.0490 (10)	0.2190 (10)	0.039 (2)	
H(161)	−0.3774 (9)	0.1389 (9)	0.2537 (8)	0.030 (2)	
H(162)	−0.4302 (9)	0.0975 (8)	0.3918 (8)	0.031 (2)	
H(171)	0.0170 (8)	0.1819 (8)	0.3589 (8)	0.022 (2)	
H(411)	0.6040 (10)	0.3780 (10)	0.8550 (10)	0.039 (3)	
H(412)	0.4720 (10)	0.4530 (10)	0.8050 (10)	0.041 (3)	
H(511)	0.7020 (10)	0.6100 (10)	0.9510 (10)	0.041 (3)	
H(512)	0.5850 (10)	0.6900 (10)	0.8910 (10)	0.039 (3)	
H(611)	−0.0920 (10)	−0.0250 (10)	−0.1190 (10)	0.043 (3)	
H(612)	−0.0550 (10)	0.1170 (10)	−0.0610 (10)	0.047 (3)	
H(711)	0.0040 (10)	0.7320 (10)	0.8750 (10)	0.049 (3)	
H(712)	−0.1410 (10)	0.7550 (10)	0.9080 (10)	0.045 (3)	
H(811)	−0.1790 (10)	0.4090 (10)	1.0130 (10)	0.044 (3)	
H(812)	−0.1370 (10)	0.4600 (10)	0.8990 (10)	0.040 (3)	
H(911)	−0.2840 (10)	0.0710 (10)	−0.0460 (10)	0.032 (3)	
H(912)	−0.3176 (9)	0.1646 (10)	0.0559 (9)	0.031 (2)	

### Atomic displacement parameters (Å^2^)

**Table:**

	*U*^11^	*U*^22^	*U*^33^	*U*^12^	*U*^13^	*U*^23^
F(1)	0.0139 (2)	0.0189 (2)	0.0175 (2)	−0.00660 (10)	0.00720 (10)	−0.00390 (10)
O(1)	0.0106 (2)	0.0146 (2)	0.0204 (2)	−0.00230 (10)	0.0016 (2)	−0.0033 (2)
O(2)	0.0155 (2)	0.0133 (2)	0.0198 (2)	−0.0025 (2)	0.0049 (2)	−0.0062 (2)
O(3)	0.0158 (2)	0.0208 (2)	0.0180 (2)	−0.0067 (2)	0.0095 (2)	−0.0096 (2)
O(41)	0.0217 (2)	0.0143 (2)	0.0567 (4)	−0.0022 (2)	0.0038 (2)	0.0000 (2)
O(51)	0.0192 (2)	0.0240 (3)	0.0313 (3)	−0.0029 (2)	−0.0015 (2)	−0.0065 (2)
O(61)	0.0268 (3)	0.0282 (3)	0.0281 (3)	0.0032 (2)	0.0103 (2)	0.0013 (2)
O(71)	0.0217 (2)	0.0201 (2)	0.0372 (3)	0.0010 (2)	0.0076 (2)	−0.0060 (2)
O(81)	0.0186 (2)	0.0314 (3)	0.0240 (3)	0.0036 (2)	0.0081 (2)	0.0086 (2)
O(91)	0.0157 (2)	0.0172 (2)	0.0161 (2)	−0.0035 (2)	0.0008 (2)	0.0002 (2)
N(1)	0.0100 (2)	0.0119 (2)	0.0094 (2)	−0.0008 (2)	0.0026 (2)	0.0000 (2)
N(2)	0.0087 (2)	0.0111 (2)	0.0125 (2)	−0.0006 (2)	0.0021 (2)	−0.0019 (2)
N(3)	0.0105 (2)	0.0127 (2)	0.0129 (2)	−0.0005 (2)	0.0007 (2)	−0.0020 (2)
C(1)	0.0107 (2)	0.0133 (2)	0.0121 (2)	−0.0001 (2)	0.0039 (2)	−0.0003 (2)
C(2)	0.0110 (2)	0.0197 (3)	0.0217 (3)	−0.0010 (2)	0.0069 (2)	−0.0033 (2)
C(3)	0.0140 (2)	0.0145 (3)	0.0196 (3)	0.0016 (2)	0.0053 (2)	−0.0019 (2)
C(4)	0.0087 (2)	0.0105 (2)	0.0107 (2)	−0.0005 (2)	0.0023 (2)	−0.0009 (2)
C(5)	0.0095 (2)	0.0107 (2)	0.0133 (2)	−0.0014 (2)	0.0028 (2)	−0.0007 (2)
C(6)	0.0097 (2)	0.0102 (2)	0.0123 (2)	−0.0016 (2)	0.0026 (2)	−0.0019 (2)
C(7)	0.0108 (2)	0.0099 (2)	0.0134 (2)	−0.0012 (2)	0.0022 (2)	−0.0012 (2)
C(8)	0.0105 (2)	0.0117 (2)	0.0119 (2)	−0.0011 (2)	0.0033 (2)	−0.0032 (2)
C(9)	0.0092 (2)	0.0106 (2)	0.0111 (2)	−0.0010 (2)	0.0024 (2)	−0.0019 (2)
C(10)	0.0101 (2)	0.0139 (2)	0.0130 (2)	−0.0022 (2)	0.0045 (2)	−0.0028 (2)
C(11)	0.0100 (2)	0.0133 (2)	0.0127 (2)	−0.0022 (2)	0.0041 (2)	−0.0018 (2)
C(12)	0.0089 (2)	0.0109 (2)	0.0112 (2)	−0.0006 (2)	0.0025 (2)	−0.0016 (2)
C(13)	0.0092 (2)	0.0122 (2)	0.0137 (2)	−0.0001 (2)	0.0026 (2)	−0.0025 (2)
C(14)	0.0111 (2)	0.0114 (2)	0.0153 (3)	0.0009 (2)	0.0006 (2)	−0.0022 (2)
C(15)	0.0096 (2)	0.0145 (3)	0.0136 (2)	−0.0019 (2)	0.0020 (2)	−0.0016 (2)
C(16)	0.0090 (2)	0.0121 (2)	0.0146 (3)	0.0007 (2)	0.0016 (2)	−0.0018 (2)
C(17)	0.0090 (2)	0.0111 (2)	0.0118 (2)	−0.0013 (2)	0.0019 (2)	−0.0016 (2)

### Geometric parameters (Å, °)

**Table:**

F(1)—C(11)	1.3504 (6)	C(2)—C(3)	1.5098 (9)
O(1)—C(7)	1.2595 (7)	C(2)—H(21)	1.070 (9)
O(2)—C(7)	1.2577 (7)	C(2)—H(22)	1.045 (10)
O(3)—C(8)	1.2520 (7)	C(3)—H(31)	1.050 (9)
O(41)—H(411)	0.931 (11)	C(3)—H(32)	1.051 (9)
O(41)—H(412)	0.965 (11)	C(4)—C(9)	1.4093 (8)
O(51)—H(511)	0.957 (11)	C(4)—C(17)	1.4125 (8)
O(51)—H(512)	0.903 (11)	C(5)—C(6)	1.3738 (8)
O(61)—H(611)	0.948 (12)	C(5)—H(51)	1.084 (9)
O(61)—H(612)	0.969 (12)	C(6)—C(7)	1.5023 (8)
O(71)—H(711)	0.941 (12)	C(6)—C(8)	1.4400 (7)
O(71)—H(712)	0.922 (11)	C(8)—C(9)	1.4650 (8)
O(81)—H(811)	0.904 (11)	C(9)—C(10)	1.4110 (7)
O(81)—H(812)	0.952 (11)	C(10)—C(11)	1.3629 (8)
O(91)—H(911)	0.919 (10)	C(10)—H(101)	1.031 (9)
O(91)—H(912)	0.943 (10)	C(11)—C(12)	1.4205 (8)
N(1)—C(1)	1.4523 (7)	C(12)—C(17)	1.3933 (7)
N(1)—C(4)	1.3940 (7)	C(13)—C(14)	1.5222 (8)
N(1)—C(5)	1.3509 (7)	C(13)—H(131)	1.086 (9)
N(2)—C(12)	1.4006 (7)	C(13)—H(132)	1.094 (9)
N(2)—C(13)	1.4663 (7)	C(14)—H(141)	1.052 (9)
N(3)—H(311)	1.026 (12)	C(14)—H(142)	1.060 (9)
N(3)—H(312)	1.025 (10)	C(15)—C(16)	1.5103 (8)
N(2)—C(16)	1.4782 (7)	C(15)—H(151)	1.061 (9)
N(3)—C(14)	1.4889 (7)	C(15)—H(152)	1.084 (10)
N(3)—C(15)	1.4910 (8)	C(16)—H(161)	1.087 (9)
C(1)—C(2)	1.5010 (8)	C(16)—H(162)	1.046 (9)
C(1)—C(3)	1.5004 (8)	C(17)—H(171)	1.079 (8)
C(1)—H(11)	1.030 (9)		
			
H(411)—O(41)—H(412)	105.7 (8)	O(3)—C(8)—C(9)	121.14 (5)
H(511)—O(51)—H(512)	104.1 (8)	C(6)—C(8)—C(9)	115.11 (5)
H(611)—O(61)—H(612)	103.5 (8)	C(4)—C(9)—C(8)	121.95 (5)
H(711)—O(71)—H(712)	107.6 (8)	C(4)—C(9)—C(10)	118.27 (5)
H(811)—O(81)—H(812)	108.2 (8)	C(8)—C(9)—C(10)	119.77 (5)
H(911)—O(91)—H(912)	106.5 (7)	C(9)—C(10)—C(11)	119.92 (5)
C(1)—N(1)—C(4)	121.37 (5)	C(9)—C(10)—H(101)	120.0 (5)
C(1)—N(1)—C(5)	119.24 (4)	C(11)—C(10)—H(101)	120.1 (5)
C(4)—N(1)—C(5)	119.30 (5)	F(1)—C(11)—C(10)	118.14 (5)
C(12)—N(2)—C(13)	116.04 (4)	F(1)—C(11)—C(12)	118.45 (5)
H(311)—N(3)—H(312)	109.3 (7)	C(10)—C(11)—C(12)	123.38 (5)
N(1)—C(1)—C(2)	118.16 (5)	N(2)—C(12)—C(11)	119.72 (5)
N(1)—C(1)—C(3)	118.70 (5)	N(2)—C(12)—C(17)	123.50 (5)
N(1)—C(1)—H(11)	113.4 (5)	C(11)—C(12)—C(17)	116.72 (5)
C(2)—C(1)—C(3)	60.40 (4)	N(2)—C(13)—C(14)	110.41 (4)
C(2)—C(1)—H(11)	118.3 (5)	N(2)—C(13)—H(131)	107.3 (4)
C(3)—C(1)—H(11)	118.1 (5)	N(2)—C(13)—H(132)	113.1 (4)
C(1)—C(2)—C(3)	59.78 (4)	C(14)—C(13)—H(131)	107.3 (4)
C(1)—C(2)—H(21)	116.0 (4)	C(14)—C(13)—H(132)	108.5 (4)
C(1)—C(2)—H(22)	114.8 (5)	H(131)—C(13)—H(132)	110.1 (6)
C(3)—C(2)—H(21)	116.1 (4)	C(13)—C(14)—H(141)	111.0 (5)
C(3)—C(2)—H(22)	117.3 (5)	C(13)—C(14)—H(142)	112.0 (4)
H(21)—C(2)—H(22)	119.0 (6)	H(141)—C(14)—H(142)	108.1 (6)
C(1)—C(3)—C(2)	59.82 (4)	N(3)—C(14)—C(13)	111.14 (5)
C(1)—C(3)—H(31)	117.8 (5)	N(3)—C(14)—H(141)	107.5 (4)
C(1)—C(3)—H(32)	115.7 (5)	N(3)—C(14)—H(142)	106.9 (4)
C(2)—C(3)—H(31)	118.9 (5)	N(3)—C(15)—C(16)	109.86 (5)
C(2)—C(3)—H(32)	116.4 (5)	N(3)—C(15)—H(151)	105.8 (5)
H(31)—C(3)—H(32)	116.3 (7)	N(3)—C(15)—H(152)	108.2 (5)
N(1)—C(4)—C(9)	119.07 (5)	C(16)—C(15)—H(151)	110.8 (5)
N(1)—C(4)—C(17)	120.24 (5)	C(16)—C(15)—H(152)	111.0 (5)
C(9)—C(4)—C(17)	120.69 (5)	H(151)—C(15)—H(152)	111.1 (7)
N(1)—C(5)—C(6)	125.09 (5)	N(2)—C(16)—C(15)	110.46 (5)
N(1)—C(5)—H(51)	116.1 (4)	N(2)—C(16)—H(161)	108.3 (4)
C(6)—C(5)—H(51)	118.8 (4)	N(2)—C(16)—H(162)	109.6 (5)
C(5)—C(6)—C(7)	117.23 (5)	C(15)—C(16)—H(161)	110.0 (4)
C(5)—C(6)—C(8)	119.45 (5)	C(15)—C(16)—H(162)	107.3 (5)
C(7)—C(6)—C(8)	123.32 (5)	H(161)—C(16)—H(162)	111.2 (6)
O(1)—C(7)—O(2)	124.85 (5)	C(4)—C(17)—C(12)	120.94 (5)
O(1)—C(7)—C(6)	117.03 (5)	C(4)—C(17)—H(171)	120.0 (4)
O(2)—C(7)—C(6)	118.12 (5)	C(12)—C(17)—H(171)	119.0 (4)
O(3)—C(8)—C(6)	123.75 (5)		
			
C(4)—N(1)—C(1)—C(2)	−139.1 (1)	N(1)—C(4)—C(17)—C(12)	177.5 (1)
C(4)—N(1)—C(1)—C(3)	−69.3 (1)	N(1)—C(4)—C(17)—H(171)	−5.7 (5)
C(1)—N(1)—C(4)—C(9)	−176.5 (1)	C(17)—C(4)—C(9)—C(8)	−178.7 (1)
C(1)—N(1)—C(4)—C(17)	3.4 (1)	C(17)—C(4)—C(9)—C(10)	2.7 (1)
C(4)—N(1)—C(1)—H(11)	76.0 (6)	C(9)—C(4)—C(17)—C(12)	−2.6 (1)
C(5)—N(1)—C(1)—C(2)	44.2 (1)	C(9)—C(4)—C(17)—H(171)	174.2 (5)
C(5)—N(1)—C(1)—C(3)	114.0 (1)	N(1)—C(5)—C(6)—C(7)	179.0 (1)
C(1)—N(1)—C(5)—C(6)	176.3 (1)	N(1)—C(5)—C(6)—C(8)	−0.6 (1)
C(5)—N(1)—C(1)—H(11)	−100.6 (6)	H(51)—C(5)—C(6)—C(7)	−0.8 (6)
C(1)—N(1)—C(5)—H(51)	−3.9 (6)	H(51)—C(5)—C(6)—C(8)	179.5 (6)
C(4)—N(1)—C(5)—C(6)	−0.4 (1)	C(5)—C(6)—C(7)—O(1)	−26.4 (1)
C(5)—N(1)—C(4)—C(9)	0.1 (1)	C(5)—C(6)—C(7)—O(2)	152.9 (1)
C(5)—N(1)—C(4)—C(17)	−179.9 (1)	C(5)—C(6)—C(8)—O(3)	−178.9 (1)
C(4)—N(1)—C(5)—H(51)	179.4 (6)	C(5)—C(6)—C(8)—C(9)	1.8 (1)
C(13)—N(2)—C(12)—C(11)	−170.1 (1)	C(8)—C(6)—C(7)—O(1)	153.3 (1)
C(12)—N(2)—C(13)—C(14)	169.4 (1)	C(8)—C(6)—C(7)—O(2)	−27.5 (1)
C(13)—N(2)—C(12)—C(17)	7.0 (1)	C(7)—C(6)—C(8)—O(3)	1.4 (1)
C(12)—N(2)—C(13)—H(131)	52.8 (5)	C(7)—C(6)—C(8)—C(9)	−177.9 (1)
C(12)—N(2)—C(13)—H(132)	−68.8 (5)	O(3)—C(8)—C(9)—C(4)	178.6 (1)
N(1)—C(1)—C(2)—C(3)	108.8 (1)	O(3)—C(8)—C(9)—C(10)	−2.9 (1)
N(1)—C(1)—C(2)—H(21)	2.4 (6)	C(6)—C(8)—C(9)—C(4)	−2.1 (1)
N(1)—C(1)—C(2)—H(22)	−142.7 (6)	C(6)—C(8)—C(9)—C(10)	176.4 (1)
N(1)—C(1)—C(3)—C(2)	−107.9 (1)	C(4)—C(9)—C(10)—C(11)	−0.3 (1)
N(1)—C(1)—C(3)—H(31)	143.1 (6)	C(4)—C(9)—C(10)—H(101)	177.3 (6)
N(1)—C(1)—C(3)—H(32)	−1.1 (6)	C(8)—C(9)—C(10)—C(11)	−178.9 (1)
C(2)—C(1)—C(3)—C(2)	0.0 (1)	C(8)—C(9)—C(10)—H(101)	−1.3 (6)
C(3)—C(1)—C(2)—C(3)	0.0 (1)	C(9)—C(10)—C(11)—F(1)	175.5 (1)
C(3)—C(1)—C(2)—H(21)	−106.5 (6)	C(9)—C(10)—C(11)—C(12)	−2.3 (1)
C(3)—C(1)—C(2)—H(22)	108.4 (6)	H(101)—C(10)—C(11)—F(1)	−2.1 (6)
C(2)—C(1)—C(3)—H(31)	−109.0 (6)	H(101)—C(10)—C(11)—C(12)	−179.9 (6)
C(2)—C(1)—C(3)—H(32)	106.9 (6)	F(1)—C(11)—C(12)—N(2)	1.9 (1)
H(11)—C(1)—C(2)—C(3)	−108.1 (6)	F(1)—C(11)—C(12)—C(17)	−175.4 (1)
H(11)—C(1)—C(2)—H(21)	145.5 (8)	C(10)—C(11)—C(12)—N(2)	179.8 (1)
H(11)—C(1)—C(2)—H(22)	0.4 (8)	C(10)—C(11)—C(12)—C(17)	2.4 (1)
H(11)—C(1)—C(3)—C(2)	108.3 (6)	N(2)—C(12)—C(17)—C(4)	−177.2 (1)
H(11)—C(1)—C(3)—H(31)	−0.7 (8)	N(2)—C(12)—C(17)—H(171)	6.0 (5)
H(11)—C(1)—C(3)—H(32)	−144.8 (8)	C(11)—C(12)—C(17)—C(4)	0.1 (1)
C(1)—C(2)—C(3)—C(1)	0.0 (1)	C(11)—C(12)—C(17)—H(171)	−176.8 (5)
C(1)—C(2)—C(3)—H(31)	107.1 (6)	N(2)—C(13)—C(14)—H(141)	175.7 (6)
C(1)—C(2)—C(3)—H(32)	−105.8 (6)	N(2)—C(13)—C(14)—H(142)	−63.4 (5)
H(21)—C(2)—C(3)—C(1)	106.2 (6)	H(131)—C(13)—C(14)—H(141)	−67.7 (7)
H(21)—C(2)—C(3)—H(31)	−146.6 (8)	H(131)—C(13)—C(14)—H(142)	53.2 (7)
H(21)—C(2)—C(3)—H(32)	0.4 (8)	H(132)—C(13)—C(14)—H(141)	51.3 (7)
H(22)—C(2)—C(3)—C(1)	−104.2 (6)	H(132)—C(13)—C(14)—H(142)	172.2 (7)
H(22)—C(2)—C(3)—H(31)	2.9 (9)	H(151)—C(15)—C(16)—H(161)	177.2 (7)
H(22)—C(2)—C(3)—H(32)	150.0 (8)	H(151)—C(15)—C(16)—H(162)	−61.7 (8)
N(1)—C(4)—C(9)—C(8)	1.2 (1)	H(152)—C(15)—C(16)—H(161)	−58.9 (8)
N(1)—C(4)—C(9)—C(10)	−177.4 (1)	H(152)—C(15)—C(16)—H(162)	62.2 (8)

### Hydrogen-bond geometry (Å, °)

**Table:**

*D*—H···*A*	*D*—H	H···*A*	*D*···*A*	*D*—H···*A*
N(3)—H(311)···O(51)^i^	1.025 (10)	2.533 (10)	3.0458 (8)	110.4 (7)
N(3)—H(311)···O(41)^ii^	1.025 (10)	1.979 (10)	2.8063 (7)	135.8 (9)
N(3)—H(312)···O(91)	1.024 (9)	1.814 (9)	2.8153 (7)	164.6 (8)
O(41)—H(412)···O(1)	0.964 (10)	1.845 (10)	2.8064 (6)	175.2 (9)
O(41)—H(411)···O(81)^iii^	0.932 (10)	1.887 (10)	2.8055 (8)	168.0 (9)
O(51)—H(511)···O(81)^iii^	0.962 (10)	1.868 (10)	2.8032 (8)	163.4 (9)
O(51)—H(512)···O(1)	0.902 (10)	1.941 (10)	2.8265 (7)	167.1 (9)
O(61)—H(611)···O(71)^iv^	0.953 (10)	1.869 (10)	2.8150 (8)	171.6 (9)
O(61)—H(612)···O(71)^v^	0.972 (10)	1.927 (11)	2.8842 (8)	167.8 (9)
O(71)—H(711)···O(2)	0.944 (10)	2.044 (10)	2.8392 (7)	140.8 (8)
O(71)—H(711)···O(3)	0.944 (10)	2.302 (10)	3.0702 (7)	138.1 (8)
O(71)—H(712)···O(51)^vi^	0.923 (10)	1.922 (10)	2.8272 (8)	166.2 (9)
O(81)—H(811)···O(2)^vii^	0.902 (11)	2.050 (10)	2.8624 (7)	149.1 (9)
O(81)—H(811)···O(3)^vii^	0.902 (11)	2.504 (10)	3.1874 (7)	132.9 (8)
O(81)—H(812)···O(3)	0.953 (10)	1.764 (10)	2.7104 (7)	171.8 (9)
O(91)—H(911)···O(61)	0.917 (10)	1.906 (10)	2.8085 (7)	167.9 (9)
O(91)—H(912)···O(2)^v^	0.942 (10)	1.791 (10)	2.7102 (6)	164.1 (8)

Symmetry codes: (i) *x*−1, *y*−1, *z*−1; (ii) −*x*, −*y*, −*z*+1; (iii) *x*+1, *y*, *z*; (iv) *x*, *y*−1, *z*−1; (v) −*x*, −*y*+1, −*z*+1; (vi) *x*−1, *y*, *z*; (vii) −*x*, −*y*+1, −*z*+2.

### Table 2 Invarioms and model compounds used for aspherical refinement of the title compound

**Table:**

Atom label	Invariom assigned	Model compound
F1	F1c	fluoromethane
O1, O2, O3	O1.5c[1.5o1c]-	acetic acid anion
O41–O91	O1h1h	water
N1, N2	N1c1c1c	trimethylamine
N3	N1c1c1h1h+	N,N-dimethylammonium
C1	C1n1c1c1h	2-aminopropane
C2, C3	C1c1c1h1h	propane
C4	C1.5c[1.5c1c]1.5c[1.5c1h]1n	o-methylaniline
C5	C1.5n[1.5c1c]1.5c[1.5c1c]1h+	N-methyl-3-methylpyridinium
C6	C1.5c[1.5n1h]1.5c[1.5c1o]1c+	3-methyl-4-hydroxypyridinium
C7	C1.5o1.5o1c-	acetic acid anion
C8	C2o1c1c	acetone
C9	C1.5c[1.5c1n]1.5c[1.5c1h]1c	o-methylaniline
C10	C1.5c[1.5c1f]1.5c[1.5c1c]1h	1-fluoro-3-methylbenzene
C11	C1.5c[1.5c1n]1.5c[1.5c1h]1f	2-fluoroaniline
C12	C1.5c[1.5c1f]1.5c[1.5c1h]1n	2-fluoroaniline
C13–C16	C1n1c1h1h	aminoethane
C17	C1.5c[1.5c1n]1.5c[1.5c1n]1h	m-phenylenediamine
H312, H322	H1n[1c1c1h]+	dimethylammonium
H11	H1c[1n1c1c]	2-aminopropane
H21–H32	H1c[1c1c1h]	propane
H51	H1c[1.5n1.5c]	pyridine
H101, H171	H1c[1.5c1.5c]	benzene
H131–H162	H1c[1n1c1h]	aminoethane
H411–H912	H1o[1h]	water
